# Accidental Puncture of the Pulmonary Artery during a Subclavian Central Venous Catheterization

**DOI:** 10.1155/2012/160847

**Published:** 2012-03-25

**Authors:** Jérôme Moriceau, Vincent Compère, Marc Bigo, Bertrand Dureuil

**Affiliations:** ^1^Department of Anesthesia and Intensive Care, Rouen University Hospital, 1 Rue de Germont, 76000 Rouen, France; ^2^Department of Anesthesia, General Hospital, 29 Avenue Pierre Mendès France, 76290 Montivilliers-Le Havre, France

## Abstract

The complications associated with central venous catheterization are common and well known. Common malplacement locations have been described in the literature. We report the case of a direct puncture of the pulmonary artery during a subclavian central venous catheterization.

## 1. Introduction

 The complications associated with subclavian central venous catheterization (CVC) ranged from 6 to 11% [1]. Specific complications associated temporally with placement of a subclavian line include hemothorax and pneumothorax, air embolism, arterial puncture, and aortic perforation [1–4]. Common malplacement locations include placement transverse to the contralateral subclavian vein or internal jugular vein [1]. We report a case of an accidental direct puncture of the pulmonary artery during a subclavian CVC.

## 2. Observation

 A cachectic 79-year-old man (weight 45 kg and height 1.65 m) with a history of ischemic heart disease and triple bypass surgery and advanced fibrosing interstitial pneumonia presented with an acute respiratory decompensation related to pneumonia (SpO_2_ at 90% via a high concentration oxygen mask; 10 L/min). The methicillin-resistant *Staphylococcus aureus* was found and required intravenous vancomycin. Because of difficulties for peripheral venous access, a left subclavian central venous catheter (plastimed Seldiflex 20 cm, Prodimed, France) was implanted. The left side was chosen because of a better skin condition. This was done by a first-term intensive care resident who had very limited technical experience (less than 20 CVC) and had not received any previous medical training. He was supervised by a senior anaesthesiologist throughout the procedure.

**Figure 1:**
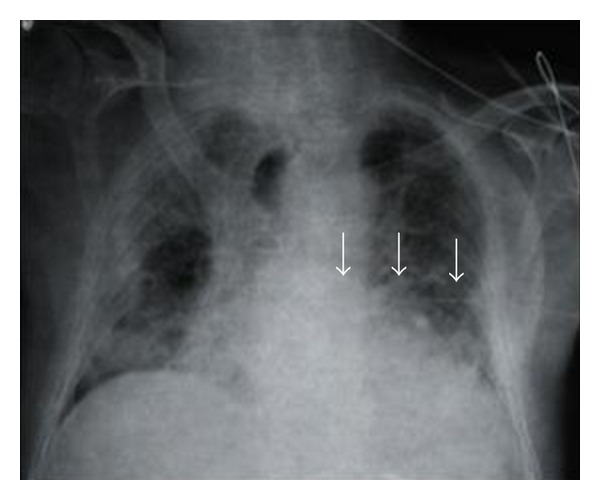
Chest radiography showing the standard aberrant course of the catheter left in the middle of the thorax (arrows).

The catheterization was performed according to the Aubaniac method at the junction of the medial third and middle third of the left clavicle, close to the lower edge [5]. Blood reflux was observed from the first puncture without encountering any technical difficulties and the catheter was introduced using the Seldinger technique. A radiological follow-up examination was performed and showed an aberrant course of the catheter in the middle of the chest without showing evidence of any pleural effusion (Figure 1).

**Figure 2:**
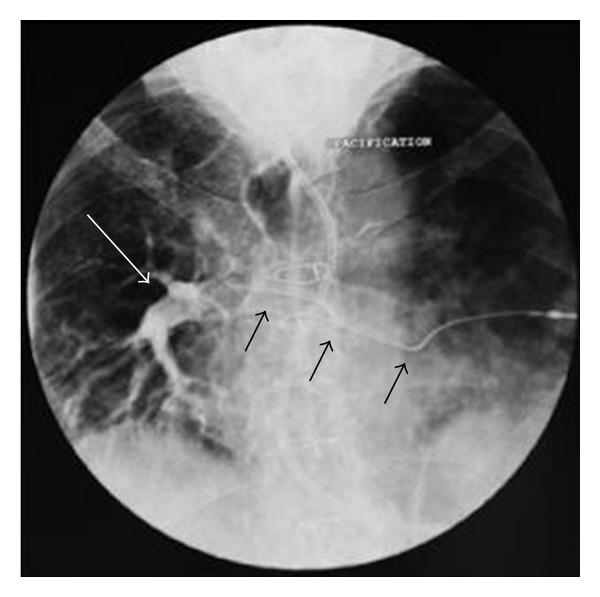
Chest radiography showing an opacification of the right pulmonary artery (white arrow) and the route of the catheter through the 2 pulmonary arteries (black arrows).

 After inspection of the patient, it was found that the puncture was made under the third rib. A radiographic control was performed by administration of contrast products. It shows the route of the catheter through the 2 pulmonary arteries (Figure 2).

 It was decided to remove the central venous catheter without further treatment and perform a CT scan a few hours later. No complication was observed. A new CVC was implanted without difficulty in the left subclavian vein. The patient died three days afterwards after worsening of the lung disease.

## 3. Discussion

 Malplacement locations of catheters are rare. Arterial or extravascular localization is also possible but rare [6, 7]. Direct catheterization of the pulmonary artery during the puncture of a subclavian CVC has never been described previously. The occurrence of this complication can largely be explained by the confusion of anatomical skin landmarks between the collarbone and the third or fourth rib. Cachexia has been one of confounding elements but it was the inexperience of the operator that played a leading role in this misjudgement. Furthermore, a likely pulmonary hypertension in this patient with advanced pulmonary fibrosis may have favoured the puncture of the pulmonary artery.

 The course after catheter removal was uneventful. This can be explained, at least partly, by the patient's previous history of cardiac surgery that could have provoked pleural adhesions and thus could have prevented the formation of a possible hemothorax and/or pneumothorax. Furthermore, the puncture was performed in a medial position and the needle could be introducing directly into the mediastinum without involving the lung parenchyma.

 Lejus and colleagues have shown that residents initially observed two or three procedures performed by seniors, but did not have theoretical lectures in 30 to 50% of cases [8]. Despite the presence of experienced anaesthesiologists during the first attempts, there was a high morbidity rate which was considered by many anaesthesiologists as a loss of benefit for the patients [8]. It was shown that above the threshold of 50 procedures made, the complication rate was decreased by 50% [9]. The introduction of a tutorial with a description of the main objectives under the supervision of seniors associated with the simulation is recommended [10, 11]. Even though the contribution of simulation in the training sequence is positive, the investment in equipment and time associated with this educational approach is made possible only at university centres with ad hoc procedures.

 Now, the ultrasound guidance to insert central venous catheter has became the gold standard. It increases the success rate, reduces the risk of complications, and induces an important reduction in the risk of failure [12, 13]. In this case report, ultrasound guidance would have shown unusual structures leading to reconsidering the landmarks initially taken.

## 4. Conclusion

 We describe an exceptional case of direct puncture of the pulmonary artery during subclavian central venous catheterization which can mainly be explained by the inexperience of the operator.

## Abbreviations

CVC: Central venous catheterization
CT: Computed tomography.
